# A Direct PCR Approach to Accelerate Analyses of Human-Associated Microbial Communities

**DOI:** 10.1371/journal.pone.0044563

**Published:** 2012-09-04

**Authors:** Gilberto E. Flores, Jessica B. Henley, Noah Fierer

**Affiliations:** 1 Cooperative Institute for Research in Environmental Sciences, University of Colorado, Boulder, Colorado, United States of America; 2 Department of Ecology and Evolutionary Biology, University of Colorado, Boulder, Colorado, United States of America; Argonne National Laboratory, United States of America

## Abstract

Since the composition of the human microbiome is highly variable both within and between individuals, researchers are increasingly reliant on high-throughput molecular approaches to identify linkages between the composition of these communities and human health. While new sequencing technologies have made it increasingly feasible to analyze large numbers of human-associated samples, the extraction of DNA from samples often remains a bottleneck in the process. Here we tested a direct PCR approach using the Extract-N-Amp Plant PCR Kit to accelerate the 16S rRNA gene-based analyses of human-associated bacterial communities, directly comparing this method to a more commonly-used approach whereby DNA is first extracted and purified from samples using a series of steps prior to PCR amplification. We used both approaches on replicate samples collected from each of five body habitats (tongue surface, feces, forehead skin, underarm skin, and forearm skin) from four individuals. With the exception of the tongue samples, there were few significant differences in the estimates of taxon richness or phylogenetic diversity obtained using the two approaches. Perhaps more importantly, there were no significant differences between the methods in their ability resolve body habitat differences or inter-individual differences in bacterial community composition and the estimates of the relative abundances of individual taxa were nearly identical with the two methods. Overall, the two methods gave very similar results and the direct PCR approach is clearly advantageous for many studies exploring the diversity and composition of human-associated bacterial communities given that large numbers of samples can be processed far more quickly and efficiently.

## Introduction

There is growing recognition that the human body harbors diverse communities of microbes and that the composition of these microbial (mostly bacterial) communities can have important effects on human health [1]–[3]. Shifts in the composition of bacterial communities found within the mouth, skin, and the gut are often associated with intra- and inter-individual variation in immune system function, resistance to opportunistic pathogens, tissue development, and metabolism [4], [5]. Research into the human microbiome and its influence on human health has long been hindered by the difficulties associated with characterizing the composition and structure of the bacterial communities. Individual samples typically harbor highly diverse bacterial communities consisting of hundreds, if not thousands, of taxa, most of these taxa can only be identified via DNA or RNA analyses and the bacterial communities found within individual body habitats are highly variable between individuals and within individuals over time [6]–[11]. Therefore, effectively quantifying the inter- and intra-individual variability in human-associated bacterial communities requires the analysis of a large number of samples using high-throughput molecular approaches.

The most commonly-used approach to determine the phylogenetic and taxonomic structure of any microbial community, including those associated with humans, is to extract and purify DNA from samples (e.g. skin swabs or fecal samples), PCR amplify the 16S rRNA gene (or a region of that gene), sequence the resulting amplicons, and then analyze the resulting sequence data. Large numbers of samples can now be analyzed in this manner given that PCR setup can readily be automated using liquid-handling robots and the increasing availability of next-generation DNA sequencing technologies [12], [13] that allow thousands of samples to be sequenced simultaneously at a very low per-sample cost [14]. Currently, the DNA extraction step is often the bottleneck in this process because most extraction protocols utilize different chemical and/or physical lysis procedures and DNA cleanup steps, many of which are very time consuming even when conducted with liquid-handling robots. Although several recent studies have evaluated a variety of DNA extraction methods to determine the most suitable for human microbiome studies [15]–[17], these studies were primarily concerned with the impact of different DNA extractions methods on community structure representation and not with expediting the extraction process.

To assess if we could more efficiently and quickly go from sample to amplified DNA suitable for high-throughput 16S rRNA gene sequencing of human-associated bacterial communities, we tested a commercially available direct PCR kit that was originally designed for extraction and amplification of plant DNA. We analyzed samples collected from human mouths (tongue), feces, and three skin locations (face, forearm and underarm) using this direct PCR approach and compared the sequence data obtained to replicate samples extracted using a more traditional protocol in order to determine if the two approaches yielded comparable information on bacterial community structure. If so, the direct PCR approach could be useful in situations where high-throughput analyses of human-associated bacterial communities are currently hindered by the time and effort required to conduct standard DNA extractions.

## Materials and Methods

### Ethics statement

Volunteers were made aware of the nature of the experiment and gave written informed consent in accordance with the sampling protocol approved by the University of Colorado Human Research Committee (protocol 0409.13).

### Sample collection

Samples were collected from five body habitats (tongue surface, feces, forehead skin, underarm skin, and forearm skin) on three men and one woman at a single point in time. These body habitats were selected as they likely represent a broad range of bacterial community types [6], [7], [18] and are sites commonly studied by microbiologists. Eight replicate samples were collected per body habitat per individual so we could compare the results obtained via the direct PCR approach with a more standard DNA extraction/purification approach (n = 4 per approach for each body habitat and individual). Samples were collected using the sterile swabbing method described previously [19] with all eight swabs held together and simultaneously brushed over the skin, tongue, or toilet paper surface. All four individuals were between the ages of 20 and 40, in good health, and had no recent history of antibiotic usage. All 160 swabs (four individuals, five body sites per individual, eight replicate samples per body habitat/individual) were stored at −20**°**C immediately after collection.

### DNA extraction and PCR amplification (standard method)

DNA was extracted from 80 of the 160 swabs using the approach described in detail in [7] and [19]. This approach involves extracting and purifying DNA from the swabs using the commercially available MoBio PowerSoil DNA Isolation Kit (MoBio Laboratories, Carlsbad, CA USA, catalog #12955), a kit that is widely used in human microbiome research [6], [7] as it consistently yields high quality, PCR-amplifiable DNA from even low biomass samples (like skin). Although numerous kits and extraction procedures have been used in human microbiome studies, all involve the basic steps of cell lysis and DNA purification prior to PCR amplification. Therefore, for the purpose of this study, we chose the MoBio PowerSoil DNA Isolation Kit to represent a “standard extraction/purification approach,” as this is what we commonly use in our laboratory and what has been adopted by such large-scale projects as The Earth Microbiome Project (http://www.earthmicrobiome.org/emp-standard-protocols/). This extraction approach involves mechanical lysis, chemical lysis, and DNA purification in a series of 32 steps following the manufacturers' instructions. All 80 samples and 16 negative controls consisting of both reagent blanks and sterile swabs were extracted in a single 96-well plate which requires approximately 6–8 h to process a full plate. For the skin and tongue samples, DNA was extracted directly from individual swab tips placed into the respective wells. For the fecal samples, the four replicate swab tips per individual were placed into a sterile, DNA-free 50 mL conical tube with 2 mL of PCR-grade water (MoBio Laboratories, Carlsbad, CA USA). The conical tube was vortexed for 30 s and the resulting fecal slurry was used to load four replicate wells in the 96-well plate with 25 µL of fecal slurry per well. The resulting DNA was then PCR amplified using a primer set (515f/806r) [20] that targets the hyper-variable V3 and V4 regions of the 16S rRNA gene. The 515f primer included the Roche 454-A FLX pyrosequencing adapter (Roche Applied Science, Branford, CT, USA) and a ‘GT’ linker sequence. The 806r primer incorporated a 12-bp error-correcting barcode sequence unique to each individual sample, the Roche 454-B FLX sequencing adapter and a ‘GG’ linker sequence. Samples were amplified in triplicate with 2 µL of forward and reverse primers (5 µM each), 10 µL of 5Prime MasterMix (5 PRIME Inc., Gaithersburg, MD, USA), 1 µL 5Prime magnesium solution and 1 µL of DNA in a total volume of 25 µL with the following cycling conditions: 35 cycles (95**°**C, 30 s; 50**°**C, 1 min; 72**°**C, 1 min) after an initial denaturation of 3 min. at 95**°**C. Amplicons from the triplicate reactions were pooled together, visualized on an agarose gel, and quantified using the PicoGreen dsDNA assay (Invitrogen, Carlsbad, CA, USA). Amplicons from all samples were pooled in equimolar concentrations into a single composite sample that was then cleaned using a single-tube MoBio UltraClean PCR Clean-up Kit (MoBio Laboratories, Carlsbad, CA USA), and sequenced at Engencore (University of South Carolina) on a Roche GS-FLX 454 automated pyrosequencer running the Titanium chemistry.

### Direct PCR amplification

**Figure 1:**
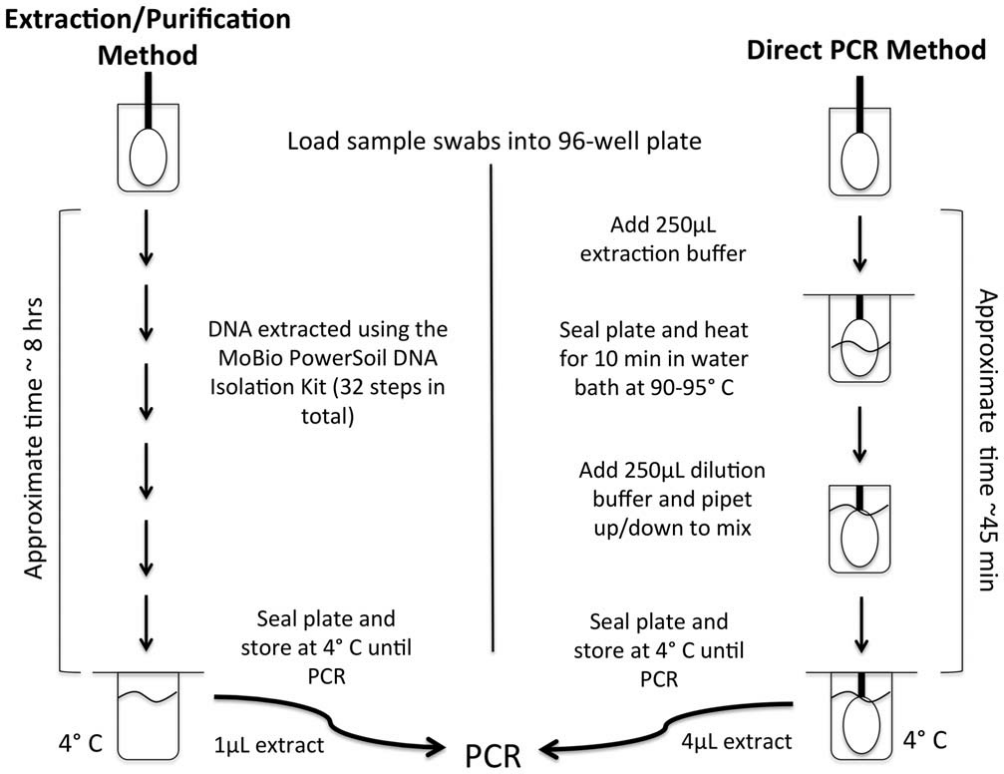
A diagram comparing the workflows used for the standard DNA extraction/purification approach and the direct PCR approach. The diagram illustrates the workflow used for the mouth and skin samples (the fecal samples were processed in a slightly different manner). See the Methods text for more detailed descriptions of these two approaches.

The other 80 samples and negative controls (again consisting of reagent blanks and sterile swabs), were prepared for sequencing using a direct PCR approach. Tongue and skin swab tips were placed directly into wells in a 2 mL 96-well Deep Well plate (Axygen Inc., Union City, CA, USA, catalog #P-DW-20-C-S-IND) along with the appropriate negative control samples. The Axygen plate was chosen for its separate well walls, which offered more uniform heating of samples as opposed to plates with shared well walls. The four replicate fecal samples from each individual were processed as described above with 25 µL of fecal slurry placed into each of four replicate wells in the 96-well plate. The plate was processed using the Extract-N-Amp Plant PCR kit (Sigma-Aldrich, Inc.) following the manufacturers' instructions except for adjusting the volume of reagents as detailed below. Wells containing fecal slurry received 100 µL of Extract-N-Amp Plant Extraction solution (catalog #E7526) and wells containing the swab tips (all negative controls, tongue, and skin samples) received 250 µL of this solution. The plate was then sealed securely with a 96 round well Impermamat Silicon Sealing Mat (Axygen Inc., Union City, CA, USA, catalog #AM-2ML-RD-IMP), heated in a water bath at 90–95**°**C for 10 minutes, followed by centrifugation for 5 min at 2500 xg. Extract-N-Amp Plant Dilution solution (catalog #D5688) was added to the wells at a 1∶1 ratio to the extraction solution and mixed gently by pipetting. The plate was resealed with the mat and stored at 4**°**C overnight. PCR was conducted in 20 µL triplicate reactions per sample using 10 µL of Extract-N-Amp Ready Mix (catalog #E3004), 1 µL of the forward and reverse primers (the same primers described above), 5 µL of PCR-grade water, and 4 µL of the Extract-N-Amp sample solutions from the 96-well plate. PCR cycling conditions were identical to those described above, as were the amplicon processing, pooling, cleaning, and sequencing methods. A flow chart comparing the two extraction protocols is shown in Figure 1.

### Sequence and statistical analyses

All sequences were processed and sorted using the default parameters in QIIME [21]. Briefly, high-quality sequences (>200 bp in length, quality score >25, exact match to barcode and primer, and containing no ambiguous characters) were clustered *de novo* into operational taxonomic units (OTUs) at 97% sequence identity using UCLUST [22]. Representative sequences of each OTU were then aligned against the Greengenes core set [23] using PyNAST [24] and assigned taxonomy with the RDP-classifier [25]. Aligned sequences were filtered using the Lane-mask and used to generate a phylogenetic tree with FastTree [26]. Average taxonomic composition was determined from replicate samples of each method.

Most (13/16) of the negative control samples associated with the direct PCR approach produced sufficient amplicon yields for sequencing. To correct for this, OTUs constituting greater than 1% of the total negative control sequences were removed from all samples prior to rarefaction and all downstream analyses. In total, 16 OTUs (out of 9,970) were removed with 75% of the sequences (4,915/6,521) belonging to the *Gammaproteobacteria*. After removal of these negative control OTUs, quality filtering of sequences, and rarefaction to 400 sequences per sample, 142 of the 160 test samples remained (Table S1). With the direct PCR protocol, none of the four replicates from the underarm of Individual C passed quality control. This was the only set of replicate samples that did not yield data with either of the approaches used.

To evaluate the suitability of the direct PCR protocol and to compare the direct PCR results to the results obtained using the extraction/purification approach, a variety of alpha and beta diversity metrics (both phylogenetic and taxonomic metrics) were calculated from the resulting sequence data. Briefly, alpha diversity is defined here as the number of taxa (OTU richness) or lineages (phylogenetic diversity, PD [27]) found in individual samples. Beta diversity is the net difference between any pair of communities: the number and abundances of taxa shared between samples (measured here using the Bray-Curtis distance metric) or the proportion of lineages shared between samples (unweighted Unifrac distance [28]). All metrics were calculated for 400 randomly selected sequences from each sample. For the alpha-diversity metrics, the average value for each sample was determined from 50 resampling events of 400 sequences per sample. Replicate samples were then averaged for each method and tested for differences between methods using a Student's t-test (within an individual) and a paired t-test (across individuals). For the beta-diversity metrics, the unweighted UniFrac distance matrix was exported from QIIME and imported into PRIMER v6 where principal coordinate analysis (PCoA) and analysis of similarity (ANOSIM) were used to visualize and statistically compare the communities observed with the different methods [29]. Bray-Curtis dissimilarities were calculated in PRIMER v6 using a normalized and square root transformed OTU table generated in QIIME. PCoA and ANOSIM were also performed on the Bray-Curtis dissimilarity matrix. Pairwise UniFrac distances and Bray-Curtis dissimilarities values for replicate samples from each individual were averaged and tested for significance using a Student's t-test to evaluate the variability between replicates within a method. All statistical tests, except ANOSIM, were performed in the R software package [30].

In addition to testing for differences in alpha and beta diversity between the two methods, we also quantified how the methods compared in their estimation of the relative abundances of specific bacterial taxa. For this, the family-level taxonomy of replicate samples from each body habitat of each individual was determined and abundances of each taxonomic group were compared between methods within individuals using a Student's t-test. Only taxonomic groups appearing in at least three pairs of samples were included in the analysis. Although dozens of taxonomic groups were compared for each body habitat, only results for the top 15 most abundant taxa are presented (Table 1).

**Table 1:** Average abundance of top 15 taxonomic groups of each body habitat from each individual observed with the direct PCR and standard extraction protocols.

		Individual A		Individual B		Individual C		Individual D	
Face		*Direct*	*Extracted*	*Direct*	*Extracted*	*Direct*	*Extracted*	*Direct*	*Extracted*
	*Staphylococcaceae* (fam., Firm.)	0.313	0.302	0.184	0.284	0.139	0.146	0.070	0.133
	*Actinomycetales* (ord., Actino.)	0.126	0.113	0.131	0.130	0.356	0.308	0.018	0.014
	*Streptococcaceae* (fam., Firm.)	0.091	0.103	0.028	0.050	0.173	0.125	0.207	0.282
	*Neisseriaceae* (fam., Beta.)	0.022	0.019	0.344	0.290	0.001	0.003	0.018	0.016
	*Corynebacteriaceae* (fam., Actino.)	0.065	0.070	0.097	0.063	0.031	0.034	0.007	0.007
	*Pasteurellaceae* (fam., Gamma.)	0.029	0.028	0.041	0.016	0.005	0.010	0.123	0.103
	*Clostridiales* family XI (fam., Firm.)	0.030	0.034	0.016	0.029	0.070	0.093	0.006	0.003
	*Moraxellaceae* (fam., Gamma.)	0.003	0.003	0.077	0.018	0.006	0.007	0.056	0.039
	*Veillonellaceae* (fam., Firm.)	0.041	0.024	0.003	0.005	0.006	0.003	0.077	0.030
	*Prevotellaceae* (fam., Bacter.)	0.019	0.013	0.008	0.001	0.004	0.010	0.063	0.054
	*Propionibacteriaceae* (fam., Actino.)	0.000	0.003	0.005	0.039	0.015	0.061	0.002	0.008
	*Lachnospiraceae* (fam., Firm.)	0.054	0.053	0.001	0.000	0.000	0.001	0.008	0.003
	*Porphyromonadaceae* (fam., Bacter.)	0.004	0.005	0.003	0.005	0.000	0.001	0.042	0.042
	*Micrococcaceae* (fam., Actino.)	0.005	0.008	0.010	0.005	0.024	0.028	0.004	0.012
	*Flavobacteriaceae* (fam., Bacter.)	0.001	0.003	0.000	0.000	0.006	0.004	0.048	0.028

		Individual A		Individual B		Individual C		Individual D	
Fecal		*Direct*	*Extracted*	*Direct*	*Extracted*	*Direct*	*Extracted*	*Direct*	*Extracted*
	*Ruminococcaceae* (fam., Firm.)	0.231	0.441	0.069	0.189	0.378	0.409	0.329	0.505
	*Lachnospiraceae* (fam., Firm.)	0.260	0.248	0.062	0.067	0.209	0.195	0.153	0.186
	*Clostridiales* (ord., Firm.)	0.129	0.083	0.059	0.029	0.173	0.138	0.113	0.071
	*Veillonellaceae* (fam., Firm.)	0.104	0.041	0.284	0.103	0.026	0.028	0.140	0.048
	*Prevotellaceae* (fam., Bacter.)	0.003	0.013	0.236	0.384	0.002	0.003	0.001	0.013
	*Bacteroidaceae* (fam., Bacter.)	0.010	0.008	0.021	0.009	0.006	0.011	0.178	0.123
	*Bifidobacteriaceae* (fam., Actino.)	0.111	0.039	0.001	0.001	0.002	0.016	0.003	0.002
	*Clostridia* (class, Firm.)	0.025	0.008	0.001	0.001	0.075	0.048	0.000	0.001
	*Bacteroidales* (ord., Bacter.)	0.000	0.002	0.054	0.088	0.000	0.001	0.004	0.000
	*Clostridiaceae* (fam., Firm.)	0.027	0.054	0.014	0.003	0.003	0.019	0.001	0.000
	*Succinivibrionaceae* (fam., Gamma.)	0.000	0.000	0.068	0.037	0.000	0.000	0.000	0.000
	*Clostridiales* family XIV (fam., Firm.)	0.014	0.018	0.001	0.012	0.013	0.019	0.001	0.019
	*Erysipelotrichaceae* (fam., Firm.)	0.008	0.008	0.004	0.021	0.001	0.007	0.028	0.006
	*Bacteroidetes* (phylum)	0.000	0.000	0.003	0.002	0.018	0.053	0.003	0.001
	*Firmicutes* (phylum)	0.004	0.003	0.002	0.009	0.037	0.017	0.000	0.002

		Individual A		Individual B		Individual C		Individual D	
Forearm		*Direct*	*Extracted*	*Direct*	*Extracted*	*Direct*	*Extracted*	*Direct*	*Extracted*
	*Actinomycetales* (ord., Actino.)	0.032	0.042	0.154	0.142	0.497	0.541	0.058	0.045
	*Staphylococcaceae* (fam., Firm.)	0.109	0.148	0.149	0.136	0.013	0.034	0.020	0.040
	*Streptococcaceae* (fam., Firm.)	0.196	0.184	0.046	0.032	0.024	0.021	0.067	0.072
	*Corynebacteriaceae* (fam., Actino.)	0.076	0.065	0.136	0.079	0.052	0.052	0.004	0.009
	*Neisseriaceae* (fam., Beta.)	0.029	0.016	0.216	0.158	0.004	0.001	0.008	0.014
	*Moraxellaceae* (fam., Gamma.)	0.022	0.026	0.018	0.010	0.035	0.033	0.078	0.079
	*Lactobacillaceae* (fam., Firm.)	0.127	0.110	0.002	0.000	0.001	0.001	0.020	0.025
	*Micrococcaceae* (fam., Actino.)	0.017	0.017	0.006	0.008	0.051	0.047	0.041	0.039
	*Veillonellaceae* (fam., Firm.)	0.033	0.028	0.010	0.008	0.002	0.000	0.084	0.039
	*Clostridiales* family XI (fam., Firm.)	0.018	0.019	0.052	0.038	0.021	0.023	0.002	0.003
	*Prevotellaceae* (fam., Bacter.)	0.021	0.026	0.037	0.048	0.003	0.000	0.025	0.003
	Unclassified Bacteria	0.014	0.007	0.013	0.029	0.011	0.003	0.043	0.043
	*Pasteurellaceae* (fam., Gamma.)	0.037	0.024	0.016	0.011	0.003	0.000	0.028	0.028
	Chloroplast (pollen)	0.028	0.046	0.004	0.006	0.007	0.003	0.029	0.016
	*Dermabacteraceae* (fam., Actino.)	0.001	0.001	0.002	0.000	0.062	0.051	0.008	0.004

		Individual A		Individual B		Individual C		Individual D	
Tongue		*Direct*	*Extracted*	*Direct*	*Extracted*	*Direct*	*Extracted*	*Direct*	*Extracted*
	*Prevotellaceae* (fam., Bacter.)	0.343	0.338	0.353	0.369	0.485	0.351	0.656	0.564
	*Veillonellaceae* (fam., Firm.)	0.338	0.284	0.213	0.164	0.215	0.194	0.228	0.296
	*Pasteurellaceae* (fam., Gamma.)	0.096	0.142	0.115	0.170	0.117	0.125	0.003	0.006
	*Streptococcaceae* (fam., Firm.)	0.145	0.099	0.108	0.080	0.096	0.098	0.055	0.056
	*Neisseriaceae* (fam., Beta.)	0.001	0.001	0.119	0.096	0.018	0.022	0.000	0.001
	*Fusobacteriaceae* (fam., Fuso.)	0.000	0.001	0.011	0.048	0.008	0.052	0.001	0.000
	*Actinomycetaceae* (fam., Actino.)	0.013	0.023	0.015	0.004	0.014	0.016	0.016	0.017
	*Leptotrichiaceae* (fam., Fuso.)	0.009	0.041	0.003	0.003	0.001	0.046	0.001	0.004
	Lactobacillales (ord., Firm.)	0.013	0.011	0.009	0.011	0.007	0.010	0.007	0.009
	Unclassified Bacteria	0.008	0.011	0.005	0.007	0.006	0.013	0.001	0.006
	*Carnobacteriaceae* (fam., Firm.)	0.008	0.008	0.011	0.006	0.005	0.006	0.001	0.004
	Actinomycetales (ord., Actino.)	0.008	0.006	0.005	0.001	0.003	0.006	0.008	0.012
	*Lachnospiraceae* (fam., Firm.)	0.006	0.011	0.001	0.003	0.002	0.013	0.004	0.002
	*Porphyromonadaceae* (fam., Bacter.)	0.001	0.000	0.009	0.015	0.004	0.006	0.000	0.000
	*Coriobacteriaceae* (fam., Actino.)	0.001	0.002	0.000	0.000	0.002	0.005	0.011	0.012

		Individual A		Individual B		Individual C		Individual D	
Underarm		*Direct*	*Extracted*	*Direct*	*Extracted*	*Direct+*	*Extracted*	*Direct*	*Extracted*
	*Staphylococcaceae* (fam., Firm.)	0.779	0.793	0.834	0.448	-	0.178	0.353	0.360
	*Corynebacteriaceae* (fam., Actino.)	0.054	0.027	0.033	0.007	-	0.397	0.003	0.000
	*Actinomycetales* (ord., Actino.)	0.004	0.002	0.012	0.006	-	0.255	0.021	0.023
	*Prevotellaceae* (fam., Bacter.)	0.003	0.018	0.006	0.127	-	0.025	0.026	0.043
	*Clostridiales* family XI (fam., Firm.)	0.062	0.048	0.018	0.010	-	0.021	0.021	0.033
	*Bacillales* (ord., Firm.)	0.021	0.031	0.024	0.014	-	0.004	0.029	0.018
	*Veillonellaceae* (fam., Firm.)	0.005	0.008	0.003	0.071	-	0.012	0.019	0.019
	Unclassified Bacteria	0.004	0.005	0.003	0.021	-	0.006	0.022	0.044
	*Streptococcaceae* (fam., Firm.)	0.005	0.008	0.004	0.026	-	0.005	0.028	0.028
	Chloroplast (pollen)	0.007	0.002	0.000	0.024	-	0.001	0.013	0.050
	*Moraxellaceae* (fam., Gamma.)	0.005	0.001	0.002	0.003	-	0.003	0.044	0.019
	*Lactobacillaceae* (fam., Firm.)	0.001	0.003	0.000	0.019	-	0.000	0.024	0.025
	*Pasteurellaceae* (fam., Gamma.)	0.001	0.013	0.000	0.016	-	0.003	0.019	0.013
	*Bacilli* (class, Firm.)	0.008	0.015	0.014	0.007	-	0.004	0.008	0.006
	*Micrococcaceae* (fam., Actino.)	0.001	0.003	0.000	0.001	-	0.011	0.018	0.028

Values in bold indicate statistical differences between the two extraction methods for that particular individual as determined by a student's t-test (p<0.01). Note that the results are presented without correcting for multiple comparisons so we are likely overestimating the number of significant differences.

+ quality sequences were not obtained for any of the four replicates from Individual C using the direct PCR protocol.

### Results and Discussion

#### Alpha diversity

**Figure 2:**
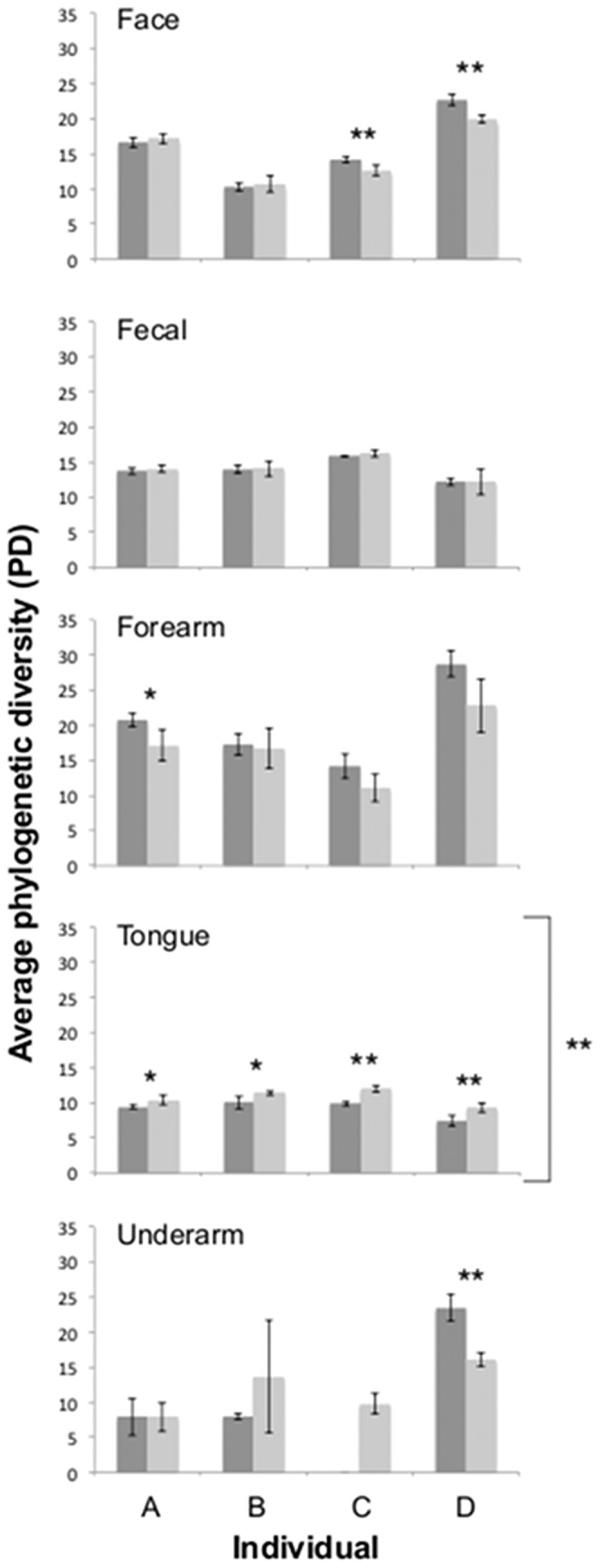
Average phylogenetic diversity observed for the communities of each body habitat of each individual using the direct PCR (dark grey) and standard extraction/purification (light grey) protocols. Bars with asterisks denote comparisons that were statistically significant within an individual (t-test, one asterisks p≤0.05, two asterisks p≤0.01). Side brackets with asterisks denote comparisons that were statistically significant across all individuals (paired t-test, p≤0.01). Error bars are ± one standard deviation.

Alpha diversity levels clearly varied across body habitats and across individuals, regardless of the metric employed (Figures 2 and S1), patterns that have been discussed in detail in other studies [7], [9], [18], [31]. Here we focus on comparisons of the estimated alpha diversity levels between the two methods, the direct PCR approach and the DNA extraction/purification approach, to determine whether they yielded similar results. With the exception of the tongue samples, there were few significant differences in the estimates of either PD levels (Figure 2) or OTU richness (Figure S1) between the two approaches. Across the four individuals, bacterial diversity on the tongue was consistently underestimated using the direct PCR approach. For the other body habitats, the two approaches yielded no consistent differences in estimated diversity across individuals, but some estimates of diversity within body habitats of specific individuals were significantly different between the two approaches, with the direct PCR approach often yielding higher estimates of diversity on skin sites than the more traditional approach. Alpha diversity estimates do appear sensitive to the method used, but the patterns across body habitats and, in most cases, the relative differences in diversity levels across individuals for a given body habitat, were consistent regardless of the method employed. We do not know how the differences between the two approaches observed here compare to the magnitude of the differences in alpha diversity estimates that are known to arise when different PCR primers [14], [32], [33], DNA extraction techniques [15]–[17], or data processing strategies [34]–[36] are employed. Alpha diversity estimates can clearly be affected by the methods employed and it is important to keep methods as consistent as possible when comparing estimates across sample sets or datasets.

#### Beta diversity

**Figure 3:**
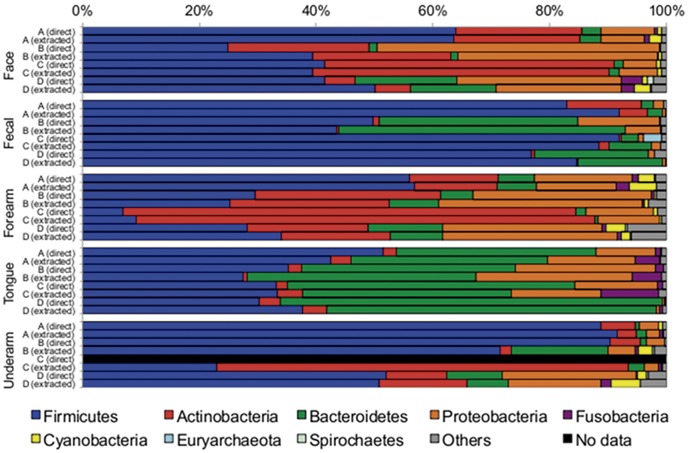
Average taxonomic composition of the various body habitats observed using both the direct and standard extraction/purification protocols. Samples are grouped by body habitat and individuals.

**Figure 4:**
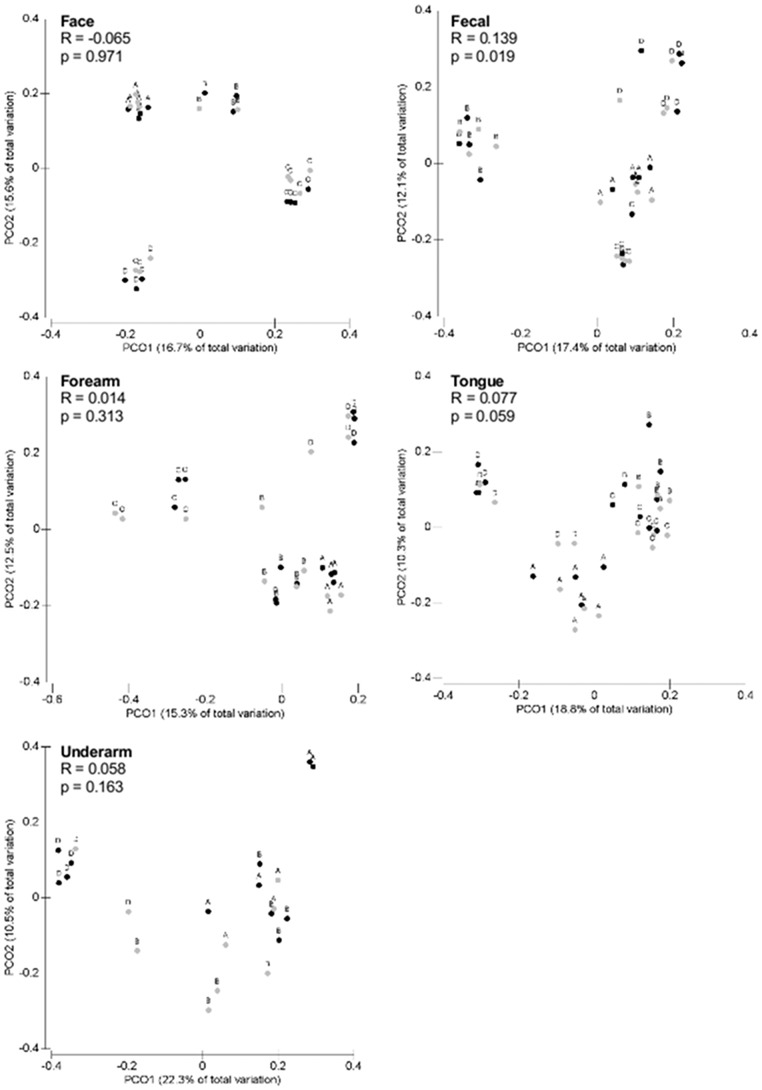
PCoA plots derived from unweighted UniFrac distances comparing the communities observed using the direct PCR (black circles) and standard extraction/purification (grey circles) protocols. Letters A–D denote individual participants. Results of the ANOSIM testing for statistical differences between methods across individuals are shown for each body habitat. Note that individual C was not included in the underarm analysis as sequences were not obtained for the direct PCR samples.

Regardless of the extraction method used, the bacterial communities primarily clustered into three groups: fecal, tongue and skin (Figure S2). The dominant taxa found in these body habitats are described in Figure 3, and the relative abundances of these groups are what we would expect based on the large number of studies that have examined human-associated bacterial communities in these body habitats (reviewed in [8], [37]. Within a given body habitat, communities clustered by individual (Figure 4, S3) regardless of the method employed, and there were no statistically significant differences between methods across individuals (p>0.01 for each body habitats; see Figures 4 and S3 for ANOSIM statistics of each body habitat). These patterns were evident when beta diversity was quantified using either a phylogenetic metric (Figure 4) or a taxonomic metric (Figure S3). These results highlight that the inter-individual differences in bacterial community composition are typically large regardless of the body habitat in question, a pattern that has been noted previously [7], [8], [19]. The differences in bacterial communities between body habitats and between individuals for a given body habitat were consistent across the two methods. If the goal of a project is to resolve inter-habitat or inter-individual differences in bacterial community composition, either method should suffice and mixing datasets obtained using the two different methods could be justified given that there were no statistically significant differences between the methods at this level of inquiry.

When we restrict our analyses to the beta diversity patterns across replicate samples collected from specific body habitats of individuals, we were able to detect significant differences between the methods for nearly all of the fecal samples, some of the tongue samples, and a few of the skin samples (Tables 2 and 3). Other studies have also observed beta diversity differences between replicate samples extracted with different DNA extraction protocols [15], [16]. The taxa driving the differences we observed between the methods are detailed in Table 1. In the gut samples, the direct PCR approach often underestimated the abundances of *Ruminococcaceae* and overestimated *Veillonellaceae* relative to the extraction/purification approach. Likewise, in the tongue samples the relative abundance of *Prevotellaceae* was, in some individuals, estimated to be higher with the direct PCR approach than the more traditional method. Although we do not know which approach is more accurate (i.e. which approach provides estimates of taxon abundances that more closely reflect true abundances), the two approaches do yield different estimates of some taxon abundances, differences that could be due to the direct PCR approach failing to release DNA from certain cell types. If the goal is to assess the intra-individual variability in bacterial communities within a given body habitat (e.g. time series studies), either method would suffice, but it is important not to mix the methods as the two methods do not yield identical estimates of taxon abundances, particularly in the fecal and tongue samples.

**Table 2:** Results of ANOSIM tests comparing the affect of extraction method on bacterial community composition (unweighted UniFrac) of each body habitat from each individual.

	Face	Fecal	Forearm	Tongue	Underarm
Individual A	−0.146	**0.583**	0.296	**0.677**	−0.056
Individual B	0.167	**0.719**	0.292	0.146	0.26
Individual C	0.094	**0.815**	0.741	**0.760**	-
Individual D	1	**0.458**	0.481	0.188	0.607

R-values in bold indicate statistically significant differences (p<0.05).

**Table 3:** Results of ANOSIM tests comparing the affect of extraction method on bacterial community structure (Bray-Curtis) of each body habitat from each individual.

	Face	Fecal	Forearm	Tongue	Underarm
Individual A	0	**1**	0.463	**0.469**	−0.13
Individual B	0	**1**	**0.323**	**0.5**	**0.385**
Individual C	0.313	**1**	0.111	**0.917**	-
Individual D	0.333	**0.927**	0.296	0.208	0.571

R-values in bold indicate statistically significant differences (p<0.05).

**Figure 5:**
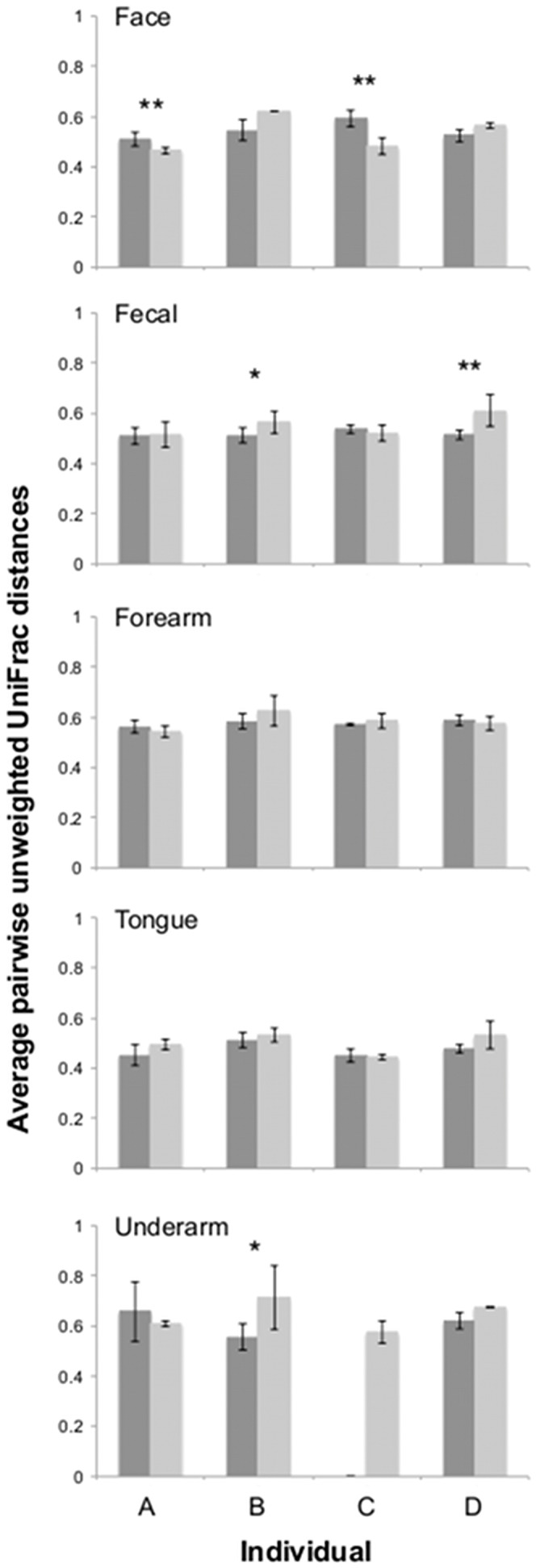
Variation of unweighted UniFrac distances of replicate samples using the direct PCR (dark grey) and standard extraction/purification (light grey) protocols. Bars with asterisks denote comparisons that were statistically significant within an individual (t-test, one asterisks p≤0.05, two asterisks p≤0.01). No statistical differences between methods across individuals were observed for any body habitats (paired t-test, p>0.05). Error bars are ± one standard deviation.

One important aspect of high-throughput sequencing is the variability in results obtained from replicate samples. Samples that are identical and are processed in an identical manner should yield very similar sequence data, but previous work has demonstrated that this is often not the case [38]. To quantify variability between replicate samples with each approach, pairwise phylogenetic distance (UniFrac) and taxonomic dissimilarity (Bray-Curtis) values were compared between replicate samples collected from the same body site and individual. With the unweighted UniFrac metric, the levels of variability between replicates were similar between the two methods with only a handful of replicate sample sets exhibiting significantly different levels of variability (Figure 5). Perhaps more importantly, one approach did not consistently yield higher variability between replicates than another, in some cases the DNA extraction/purification approach actually resulted in greater variability between replicate samples. Similar patterns were observed using Bray-Curtis dissimilarities although the direct PCR communities from the fecal and tongue samples were more variable across individuals (Figure S4). Regardless of the approach used, replicate samples never yielded identical sequence data (a point that should be considered when designing studies) and this variability between replicates was not consistently higher with one approach than the other.

### Conclusion

The direct approach is far faster – going from sample to amplicons suitable for sequencing was at least 6 to 8 times faster with the direct PCR approach than the DNA extraction/purification approach (Figure 1). Although there are likely DNA extraction methods that are faster than the MoBio extraction kit used here, we know of no stand-alone technique for DNA extraction that yields PCR-amplifiable DNA from such a high number of human-associated samples as quickly as the direct PCR approach used here. The direct PCR approach is also likely cheaper given the considerable labor savings. For studies where it is critical to process a large number of human-associated samples quickly and efficiently for PCR-based bacterial community analyses, the direct PCR approach described here is clearly a useful tool given that the results are, in most cases, directly comparable to the results obtained using the more time consuming standard approach.

Of course, the direct PCR approach does have its limitations that render it unsuitable (or at least less suitable) for certain applications and study designs. The direct PCR approach is likely not appropriate for shotgun metagenomic analyses and, if a given sample is to be used for multiple PCR-based assays, it would probably be useful to extract DNA using a standard approach. However, we have successfully conducted multiple PCR analyses off of Extract-N-Amp dilution solutions stored at 4°C for up to 20 weeks (data not shown), but this is not likely a long term storage option. If DNA needs to be stored for extended periods of time for downstream analyses, the more standard extraction approach would likely be preferable. Also, we do not know how the direct PCR approach would work for other types of PCR-based analyses (e.g. quantitative PCR analyses targeting specific pathogens). Nevertheless, as studies are increasingly moving to very large sample numbers and amplicon sequencing becomes ever faster and cheaper, the direct PCR approach is clearly a valuable option for high-throughput studies examining the structure and diversity of human-associated bacterial communities.

### Supporting Information

Figure S1 **Average number of OTUs observed for each body habitat of each individual using the direct PCR (dark grey) and standard extraction/purification (light grey) protocols.** Bars with asterisks denote comparisons that were statistically significant within an individual (t-test, one asterisks p≤0.05, two asterisks p≤0.01). Side brackets with asterisks denote comparisons that were statistically significant across all individuals (paired t-test, p≤0.01). Error bars are ± one standard deviation.
(TIF)

Figure S2 **PCoA plots illustrating differences in community composition across body habitats (a and c) and no differences based on which protocol was used across individuals (b and d).** Plots **a** and **b** are based on unweighted UniFrac distances while **c** and **d** are based on Bray-Curtis dissimilarity values. Results of ANOSIM tests are presented in the bottom right of each plot.
(TIF)

Figure S3 **PCoA plots derived from Bray-Curtis dissimilarities comparing the communities observed using the direct PCR (black circles) and standard extraction/purification (grey circles) protocols.** Letters A-D denote individual participants. Results of the ANOSIM testing for statistical differences between methods across individuals are shown for each body habitat. Note that individual C was not included in the underarm analysis as sequences were not obtained for the direct PCR samples.
(TIF)

Figure S4 **Variation of Bray-Curtis dissimilarities of replicate samples using the direct PCR (dark grey) and standard extraction/purification (light grey) protocols.** Bars with asterisks denote comparisons that were statistically significant within an individual (t-test, one asterisks p≤0.05, two asterisks p≤0.01). Only communities from the tongue showed differences between methods across individuals (paired t-test, p≤0.05). Error bars are ± one standard deviation.
(TIF)

Table S1 **Samples used to evaluate the suitability of the direct PCR protocol for use in high-throughput 16S rRNA gene surveys of the human microbiome.** Note that the before subtraction column refers to the number of sequences before removal of negative control OTUs present as 1% or greater of total negative control sequences. For all downstream analyses, samples were rarefied to 400 sequences per sample using sequences after subtraction.
(DOC)
