# Supplementary figures and images for: A Direct PCR Approach to Accelerate Analyses of Human-Associated Microbial Communities
Source: PLoS One. 2012 Sep 4;7(9):e44563. doi: 10.1371/journal.pone.0044563 (PMC3433448; doi:10.1371/journal.pone.0044563)

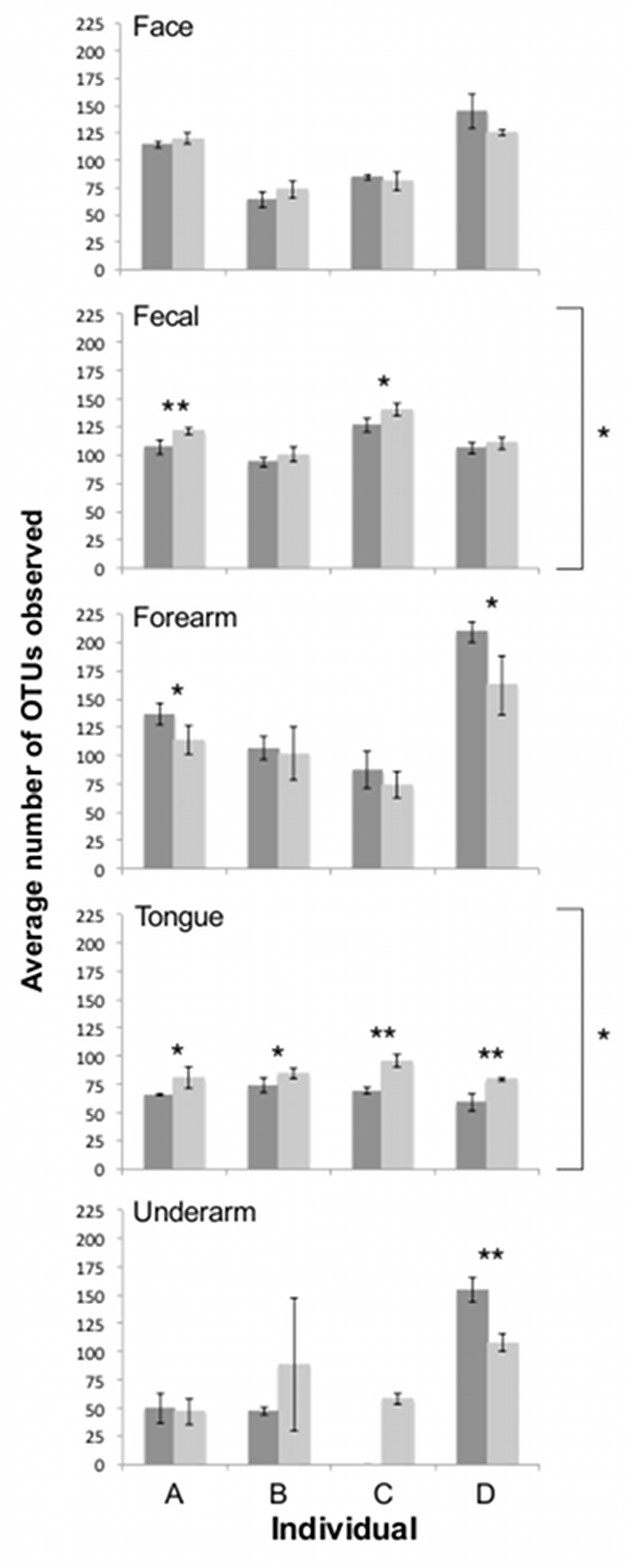

Supplement: Figure S1 — Average number of OTUs observed for each body habitat of each individual using the direct PCR (dark grey) and standard extraction/purification (light grey) protocols. Bars with asterisks denote comparisons that were statistically significant within an individual (t-test, one asterisks p≤0.05, two asterisks p≤0.01). Side brackets with asterisks denote comparisons that were statistically significant across all individuals (paired t-test, p≤0.01). Error bars are ± one standard deviation. (TIF) [file pone.0044563.s001.tif]

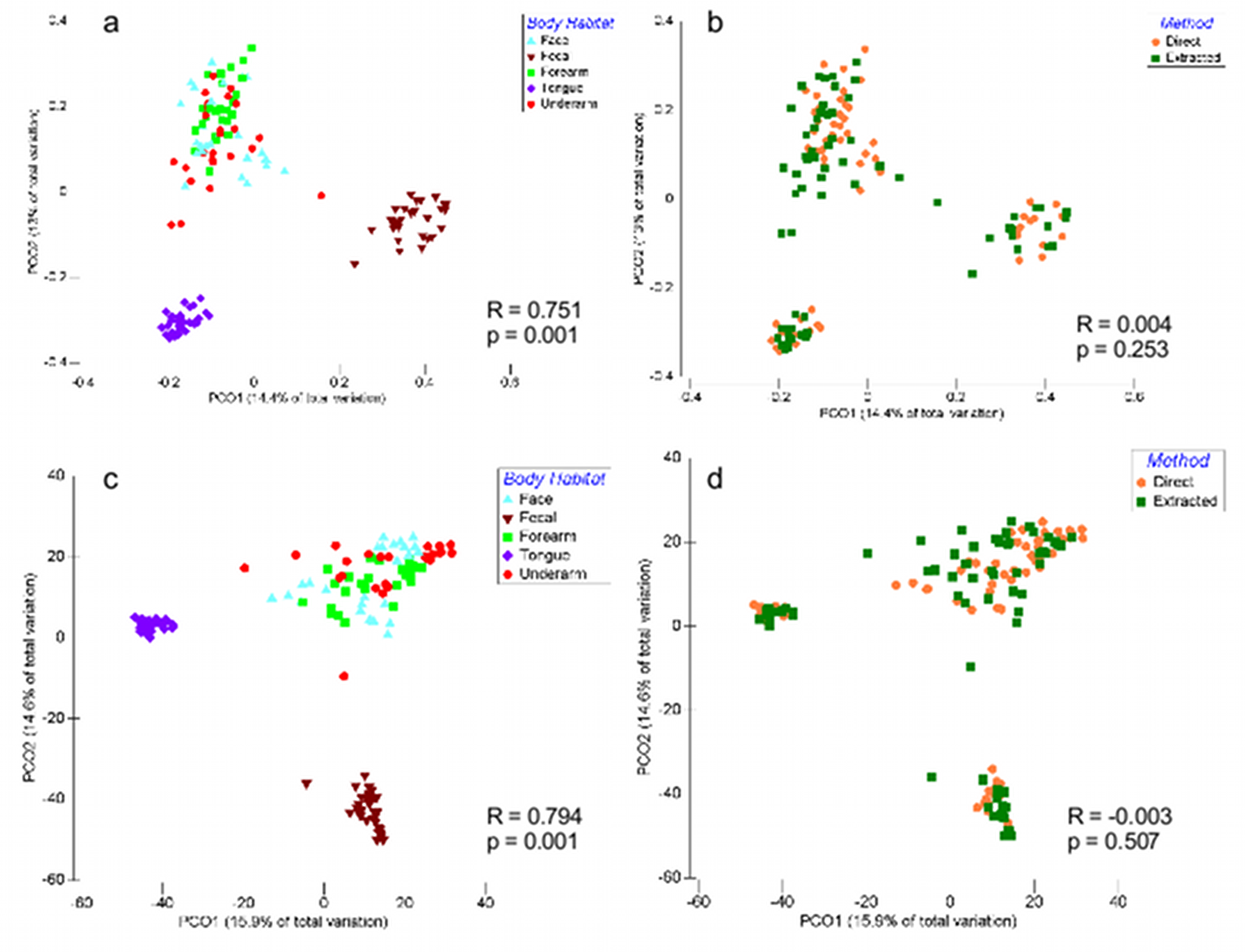

Supplement: Figure S2 — PCoA plots illustrating differences in community composition across body habitats (a and c) and no differences based on which protocol was used across individuals (b and d). Plots a and b are based on unweighted UniFrac distances while c and d are based on Bray-Curtis dissimilarity values. Results of ANOSIM tests are presented in the bottom right of each plot. (TIF) [file pone.0044563.s002.tif]

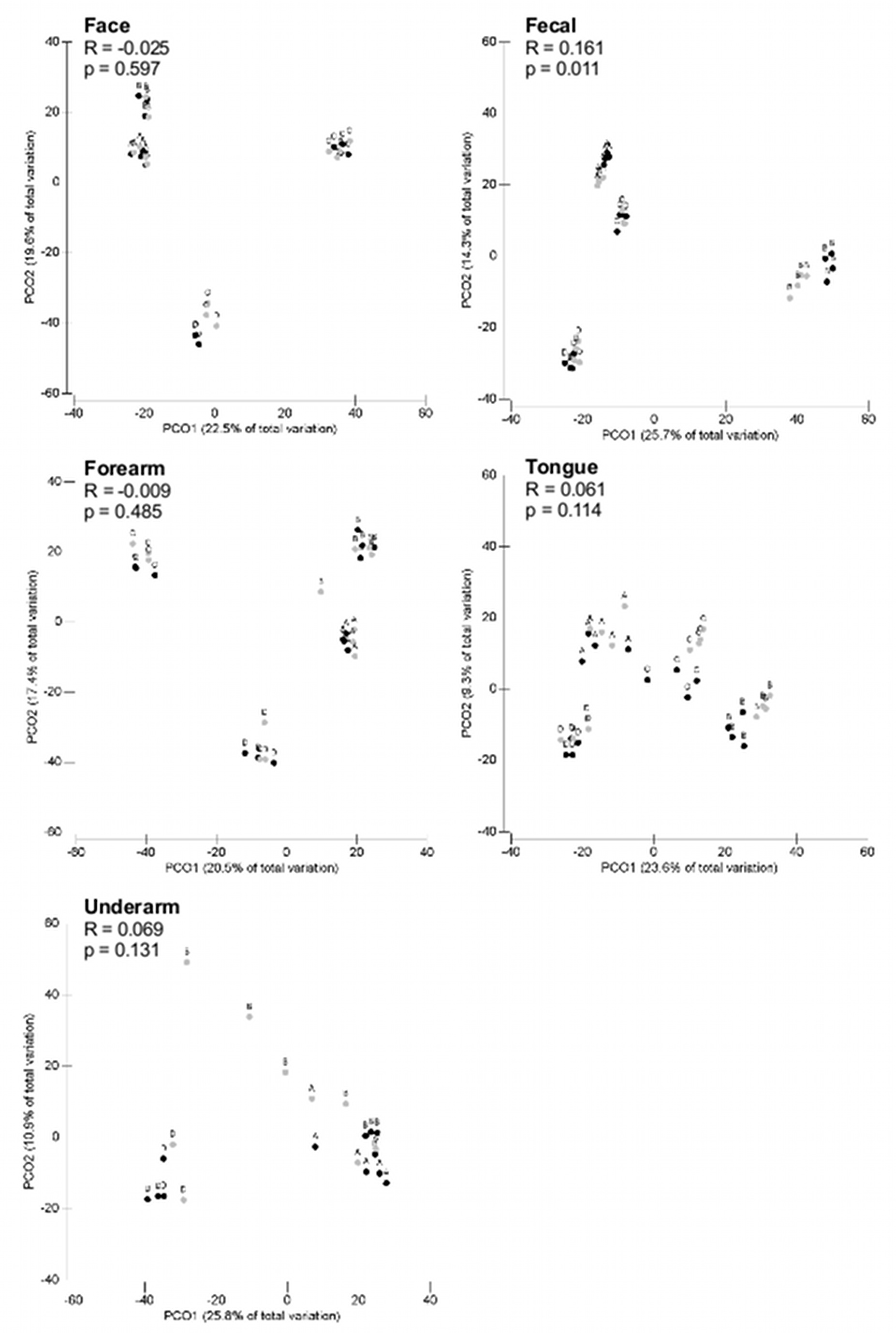

Supplement: Figure S3 — PCoA plots derived from Bray-Curtis dissimilarities comparing the communities observed using the direct PCR (black circles) and standard extraction/purification (grey circles) protocols. Letters A-D denote individual participants. Results of the ANOSIM testing for statistical differences between methods across individuals are shown for each body habitat. Note that individual C was not included in the underarm analysis as sequences were not obtained for the direct PCR samples. (TIF) [file pone.0044563.s003.tif]

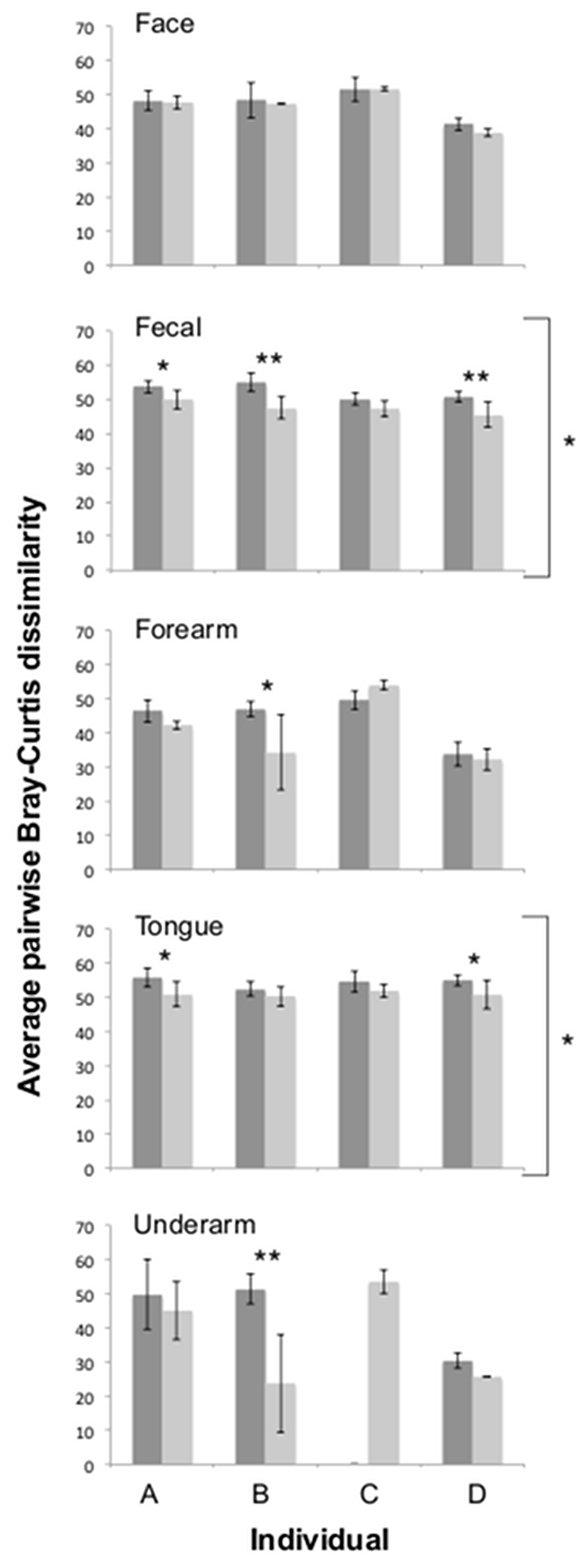

Supplement: Figure S4 — Variation of Bray-Curtis dissimilarities of replicate samples using the direct PCR (dark grey) and standard extraction/purification (light grey) protocols. Bars with asterisks denote comparisons that were statistically significant within an individual (t-test, one asterisks p≤0.05, two asterisks p≤0.01). Only communities from the tongue showed differences between methods across individuals (paired t-test, p≤0.05). Error bars are ± one standard deviation. (TIF) [file pone.0044563.s004.tif]
