# Supplementary material for: A Direct PCR Approach to Accelerate Analyses of Human-Associated Microbial Communities
Source: PLoS One. 2012 Sep 4;7(9):e44563. doi: 10.1371/journal.pone.0044563 (PMC3433448; doi:10.1371/journal.pone.0044563)
Supplement: Table S1 — Samples used to evaluate the suitability of the direct PCR protocol for use in high-throughput 16S rRNA gene surveys of the human microbiome. Note that the before subtraction column refers to the number of sequences before removal of negative control OTUs present as 1% or greater of total negative control sequences. For all downstream analyses, samples were rarefied to 400 sequences per sample using sequences after subtraction. (DOC) [file pone.0044563.s005.doc]

Table S1. Samples used to evaluate the suitability of the direct PCR protocol for use in high-throughput 16S rRNA gene surveys of the human microbiome. Note that the before subtraction column refers to the number of sequences before removal of negative control OTUs present as 1% or greater of total negative control sequences. For all downstream analyses, samples were rarefied to 400 sequences per sample using sequences after subtraction.

| **Sample name** | **Individual** | **Body habitat** | **Method** | **Before subtraction** | **After subtraction** |
| --- | --- | --- | --- | --- | --- |
| DirA.F1 | A | Face | Direct | 1,302 | 1,279 |
| DirA.F2 | A | Face | Direct | 1,076 | 1,052 |
| DirA.F3 | A | Face | Direct | 483 | 477 |
| DirA.F4 | A | Face | Direct | 1,237 | 1,194 |
| DirA.L1 | A | Fecal | Direct | 1,206 | 1,206 |
| DirA.L2 | A | Fecal | Direct | 1,443 | 1,443 |
| DirA.L3 | A | Fecal | Direct | 1,343 | 1,343 |
| DirA.L4 | A | Fecal | Direct | 1,821 | 1,820 |
| DirA.R1 | A | Forearm | Direct | 1,698 | 1,559 |
| DirA.R2 | A | Forearm | Direct | 1,211 | 1,044 |
| DirA.R3 | A | Forearm | Direct | 1,850 | 1,719 |
| DirA.R4 | A | Forearm | Direct | 1,564 | 1,480 |
| DirA.T1 | A | Tongue | Direct | 1,213 | 1,213 |
| DirA.T2 | A | Tongue | Direct | 1,670 | 1,670 |
| DirA.T3 | A | Tongue | Direct | 1,834 | 1,834 |
| DirA.T4 | A | Tongue | Direct | 671 | 671 |
| DirA.U1 | A | Underarm | Direct | 1,517 | 1,299 |
| DirA.U2 | A | Underarm | Direct | 1,301 | 1,288 |
| DirA.U3 | A | Underarm | Direct | 1,426 | 1,354 |
| DirA.U4 | A | Underarm | Direct | 1,607 | 1,583 |
| ExtA.F1 | A | Face | Extracted | 2,069 | 2,049 |
| ExtA.F2 | A | Face | Extracted | 2,274 | 2,243 |
| ExtA.F3 | A | Face | Extracted | 2,244 | 2,218 |
| ExtA.F4 | A | Face | Extracted | 1,680 | 1,660 |
| ExtA.L1 | A | Fecal | Extracted | 2,528 | 2,528 |
| ExtA.L2 | A | Fecal | Extracted | 2,752 | 2,751 |
| ExtA.L3 | A | Fecal | Extracted | 1,371 | 1,371 |
| ExtA.L4 | A | Fecal | Extracted | 2,560 | 2,560 |
| ExtA.R1 | A | Forearm | Extracted | 213 | 204 |
| ExtA.R2 | A | Forearm | Extracted | 1,898 | 1,859 |
| ExtA.R3 | A | Forearm | Extracted | 514 | 505 |
| ExtA.R4 | A | Forearm | Extracted | 880 | 861 |
| ExtA.T1 | A | Tongue | Extracted | 2,344 | 2,344 |
| ExtA.T2 | A | Tongue | Extracted | 673 | 673 |
| ExtA.T3 | A | Tongue | Extracted | 2,146 | 2,146 |
| ExtA.T4 | A | Tongue | Extracted | 2,544 | 2,544 |
| ExtA.U1* | A | Underarm | Extracted | - | - |
| ExtA.U2 | A | Underarm | Extracted | 2,010 | 2,000 |
| ExtA.U3 | A | Underarm | Extracted | 1,681 | 1,676 |
| ExtA.U4 | A | Underarm | Extracted | 1,734 | 1,729 |
| DirB.F1 | B | Face | Direct | 361 | 361 |
| DirB.F2 | B | Face | Direct | 1,232 | 1,218 |
| DirB.F3 | B | Face | Direct | 855 | 848 |
| DirB.F4 | B | Face | Direct | 948 | 942 |
| DirB.L1 | B | Fecal | Direct | 1,370 | 1,349 |
| DirB.L2 | B | Fecal | Direct | 1,198 | 1,181 |
| DirB.L3 | B | Fecal | Direct | 1,580 | 1,562 |
| DirB.L4 | B | Fecal | Direct | 1,392 | 1,374 |
| DirB.R1 | B | Forearm | Direct | 1,346 | 1,277 |
| DirB.R2 | B | Forearm | Direct | 1,647 | 1,606 |
| DirB.R3 | B | Forearm | Direct | 623 | 595 |
| DirB.R4 | B | Forearm | Direct | 1,161 | 1,114 |
| DirB.T1 | B | Tongue | Direct | 1,497 | 1,497 |
| DirB.T2 | B | Tongue | Direct | 1,070 | 1,070 |
| DirB.T3 | B | Tongue | Direct | 1,449 | 1,449 |
| DirB.T4 | B | Tongue | Direct | 1,468 | 1,468 |
| DirB.U1 | B | Underarm | Direct | 1,585 | 1,535 |
| DirB.U2 | B | Underarm | Direct | 422 | 408 |
| DirB.U3 | B | Underarm | Direct | 1,098 | 1,059 |
| DirB.U4 | B | Underarm | Direct | 1,759 | 1,712 |
| ExtB.F1 | B | Face | Extracted | 1,835 | 1,827 |
| ExtB.F2 | B | Face | Extracted | 2 | 2 |
| ExtB.F3 | B | Face | Extracted | 155 | 154 |
| ExtB.F4 | B | Face | Extracted | 1,689 | 1,678 |
| ExtB.L1 | B | Fecal | Extracted | 1,819 | 1,816 |
| ExtB.L2 | B | Fecal | Extracted | 744 | 742 |
| ExtB.L3 | B | Fecal | Extracted | 519 | 516 |
| ExtB.L4 | B | Fecal | Extracted | 3,427 | 3,411 |
| ExtB.R1 | B | Forearm | Extracted | 1,553 | 1,528 |
| ExtB.R2 | B | Forearm | Extracted | 1,322 | 1,307 |
| ExtB.R3 | B | Forearm | Extracted | 916 | 848 |
| ExtB.R4 | B | Forearm | Extracted | 537 | 529 |
| ExtB.T1 | B | Tongue | Extracted | 2,377 | 2,377 |
| ExtB.T2 | B | Tongue | Extracted | 2,300 | 2,300 |
| ExtB.T3 | B | Tongue | Extracted | 2,429 | 2,429 |
| ExtB.T4 | B | Tongue | Extracted | 3,180 | 3,180 |
| ExtB.U1 | B | Underarm | Extracted | 423 | 423 |
| ExtB.U2 | B | Underarm | Extracted | 681 | 679 |
| ExtB.U3 | B | Underarm | Extracted | 930 | 929 |
| ExtB.U4 | B | Underarm | Extracted | 980 | 980 |
| DirC.F1 | C | Face | Direct | 1,278 | 1,244 |
| DirC.F2 | C | Face | Direct | 824 | 814 |
| DirC.F3 | C | Face | Direct | 1,397 | 1,370 |
| DirC.F4 | C | Face | Direct | 834 | 815 |
| DirC.L1 | C | Fecal | Direct | 1,753 | 1,746 |
| DirC.L2 | C | Fecal | Direct | 68 | 67 |
| DirC.L3 | C | Fecal | Direct | 2,115 | 2,109 |
| DirC.L4 | C | Fecal | Direct | 1,471 | 1,463 |
| DirC.R1 | C | Forearm | Direct | 986 | 908 |
| DirC.R2 | C | Forearm | Direct | 998 | 915 |
| DirC.R3* | C | Forearm | Direct | - | - |
| DirC.R4 | C | Forearm | Direct | 1,259 | 1,126 |
| DirC.T1 | C | Tongue | Direct | 1,717 | 1,717 |
| DirC.T2 | C | Tongue | Direct | 1,649 | 1,649 |
| DirC.T3 | C | Tongue | Direct | 1,725 | 1,725 |
| DirC.T4 | C | Tongue | Direct | 889 | 889 |
| DirC.U1 | C | Underarm | Direct | 187 | 64 |
| DirC.U2 | C | Underarm | Direct | 195 | 81 |
| DirC.U3 | C | Underarm | Direct | 215 | 78 |
| DirC.U4 | C | Underarm | Direct | 279 | 87 |
| ExtC.F1 | C | Face | Extracted | 1,549 | 1,538 |
| ExtC.F2 | C | Face | Extracted | 1,640 | 1,633 |
| ExtC.F3 | C | Face | Extracted | 2,054 | 2,039 |
| ExtC.F4 | C | Face | Extracted | 1,631 | 1,614 |
| ExtC.L1 | C | Fecal | Extracted | 2,152 | 2,147 |
| ExtC.L2 | C | Fecal | Extracted | 2,248 | 2,231 |
| ExtC.L3 | C | Fecal | Extracted | 2,544 | 2,534 |
| ExtC.L4 | C | Fecal | Extracted | 2,420 | 2,413 |
| ExtC.R1 | C | Forearm | Extracted | 995 | 988 |
| ExtC.R2 | C | Forearm | Extracted | 2 | 2 |
| ExtC.R3 | C | Forearm | Extracted | 1,178 | 1,166 |
| ExtC.R4 | C | Forearm | Extracted | 808 | 797 |
| ExtC.T1 | C | Tongue | Extracted | 2,304 | 2,304 |
| ExtC.T2 | C | Tongue | Extracted | 2,642 | 2,642 |
| ExtC.T3 | C | Tongue | Extracted | 2,199 | 2,199 |
| ExtC.T4 | C | Tongue | Extracted | 2,532 | 2,532 |
| ExtC.U1 | C | Underarm | Extracted | 1,062 | 1,060 |
| ExtC.U2 | C | Underarm | Extracted | 1,920 | 1,917 |
| ExtC.U3 | C | Underarm | Extracted | 762 | 761 |
| ExtC.U4 | C | Underarm | Extracted | 1,475 | 1,472 |
| DirD.F1 | D | Face | Direct | 6 | 6 |
| DirD.F2 | D | Face | Direct | 968 | 942 |
| DirD.F3 | D | Face | Direct | 1,262 | 1,225 |
| DirD.F4 | D | Face | Direct | 1,483 | 1,449 |
| DirD.L1 | D | Fecal | Direct | 2,281 | 2,280 |
| DirD.L2 | D | Fecal | Direct | 1,401 | 1,401 |
| DirD.L3 | D | Fecal | Direct | 1,854 | 1,854 |
| DirD.L4 | D | Fecal | Direct | 1,767 | 1,765 |
| DirD.R1 | D | Forearm | Direct | 69 | 62 |
| DirD.R2 | D | Forearm | Direct | 1,391 | 1,200 |
| DirD.R3 | D | Forearm | Direct | 1,069 | 910 |
| DirD.R4 | D | Forearm | Direct | 740 | 653 |
| DirD.T1 | D | Tongue | Direct | 1,452 | 1,452 |
| DirD.T2 | D | Tongue | Direct | 546 | 546 |
| DirD.T3 | D | Tongue | Direct | 754 | 754 |
| DirD.T4 | D | Tongue | Direct | 1,365 | 1,365 |
| DirD.U1 | D | Underarm | Direct | 1,020 | 839 |
| DirD.U2 | D | Underarm | Direct | 1,165 | 990 |
| DirD.U3 | D | Underarm | Direct | 1,385 | 1,122 |
| DirD.U4 | D | Underarm | Direct | 761 | 607 |
| ExtD.F1 | D | Face | Extracted | 103 | 101 |
| ExtD.F2 | D | Face | Extracted | 2,214 | 2,180 |
| ExtD.F3 | D | Face | Extracted | 669 | 665 |
| ExtD.F4 | D | Face | Extracted | 1,499 | 1,477 |
| ExtD.L1 | D | Fecal | Extracted | 2,878 | 2,878 |
| ExtD.L2 | D | Fecal | Extracted | 3,672 | 3,671 |
| ExtD.L3 | D | Fecal | Extracted | 3,348 | 3,348 |
| ExtD.L4 | D | Fecal | Extracted | 3,510 | 3,510 |
| ExtD.R1 | D | Forearm | Extracted | 1,909 | 1,835 |
| ExtD.R2 | D | Forearm | Extracted | 1,239 | 1,153 |
| ExtD.R3 | D | Forearm | Extracted | 63 | 58 |
| ExtD.R4 | D | Forearm | Extracted | 464 | 427 |
| ExtD.T1 | D | Tongue | Extracted | 3,006 | 3,006 |
| ExtD.T2 | D | Tongue | Extracted | 1,928 | 1,928 |
| ExtD.T3 | D | Tongue | Extracted | 2,833 | 2,833 |
| ExtD.T4 | D | Tongue | Extracted | 2,342 | 2,342 |
| ExtD.U1 | D | Underarm | Extracted | 695 | 656 |
| ExtD.U2 | D | Underarm | Extracted | 266 | 261 |
| ExtD.U3 | D | Underarm | Extracted | 627 | 599 |
| ExtD.U4* | D | Underarm | Extracted | - | - |
| DirB1 | Blank | - | Direct | 82 | 29 |
| DirB2 | Blank | - | Direct | 226 | 50 |
| DirB3 | Blank | - | Direct | 93 | 18 |
| DirB4 | Blank | - | Direct | 227 | 64 |
| DirB5 | Blank | - | Direct | 228 | 59 |
| DirB6 | Blank | - | Direct | 146 | 27 |
| DirB7 | Blank | - | Direct | 82 | 32 |
| DirB8 | Blank | - | Direct | 233 | 73 |
| DirB9 | Blank | - | Direct | 170 | 65 |
| DirB10 | Blank | - | Direct | 139 | 36 |
| DirB11 | Blank | - | Direct | 230 | 49 |
| DirB12 | Blank | - | Direct | 256 | 84 |
| DirBS | Blank | - | Direct | 158 | 46 |
| ExtB1 | Blank | - | Extracted | 237 | 237 |
| ExtB2 | Blank | - | Extracted | 1 | 0 |
| ExtB3 | Blank | - | Extracted | 1,098 | 1,096 |
| ExtB4 | Blank | - | Extracted | 2,684 | 2,684 |
| NT.Control1 | PCR | - | - | 261 | 124 |
| NT.Control2 | PCR | - | - | 264 | 148 |
| NT.Control3 | PCR | - | - | 409 | 274 |
| **Total # of sequences from direct PCR** | | | | **97,316** | **91,620** |
| **Total # of sequences from standard extraction** | | | | **133,335** | **132,510** |
| **Total # of sequences** | | | | **230,651** | **224,130** |

*denotes samples not used in the study due to failed amplification (n=3).
